# Cocaine Induced Biliary Tree Obstruction in a Middle-Aged Male

**DOI:** 10.7759/cureus.20458

**Published:** 2021-12-16

**Authors:** Karlbuto Alexandre, Oyindayo Hassan, James Hebden, John M Barnwell

**Affiliations:** 1 Department of Surgery, Detroit Medical Center - Sinai Grace Hospital, Detroit, USA; 2 Department of Medicine, Meharry Medical College, Nashville, USA; 3 Department of Emergency Medicine, Beaumont Health - Farmington Hills, Farmington Hills, USA

**Keywords:** s: cholangiocarcinoma, cholangiocarcinoma, lymphadeopathy, surgery general, biliary stricture, cocaine use, malignant biliary obstruction, biliary obstruction

## Abstract

A sixty-two-year-old male with a history of extensive crack cocaine use and cholecystectomy presented to the emergency department with abdominal pain, nausea, vomiting, and urobilia. The physical exam revealed moderate epigastric tenderness without scleral icterus or jaundice. The patient's total bilirubin was elevated at 5.2, and his direct bilirubin was 3.7. A computed tomography angiogram (CTA) of the abdomen and pelvis subsequently showed a 3.1 x 2.8 cm mass compressing porta hepatis. A magnetic resonance cholangiopancreatography (MRCP) showed a 4.9 x 3.0 cm mass at the porta hepatis with corresponding biliary duct obstruction at that level. An endoscopic retrograde cholangiopancreatography (ERCP) was performed with stent placement and brush biopsy, which showed predominantly benign ductal epithelium with rare, atypical cells and stenosis of the proximal common bile duct suggestive of cholangiocarcinoma. Cytology was performed on the ductal fluid and was also negative. The carbohydrate antigen (CA) 19-9 level at that time was 94.3. We discussed the possibility of performing surgery as an inpatient, but the patient had various psychosocial issues, which prompted a psychiatric evaluation. He subsequently had an internal-external biliary drain placed. The patient was discharged with plans to obtain an endoscopic ultrasound as an outpatient. He was admitted and discharged several times over the span of six months for various issues. He received an endoscopic ultrasound (EUS) at a surrounding hospital. The results were inconclusive, and a repeat EUS was recommended. On the last admission to the hospital for abdominal pain, a CT scan showed no biliary tree obstruction, which was further confirmed with an MRCP. The internal-external biliary drain was removed without recurrence of hyperbilirubinemia. We suspect that the patient's initial symptoms and radiographic findings of a biliary tree mass may have been induced by extrinsic compression secondary to lymphadenopathy caused by an adulterant used in the cutting process of abused cocaine. This is a rare occurrence that has not been described in the literature. There are associations of cocaine use to pulmonary hilar lymphadenopathy, but not biliary lymphadenopathy. We strongly suspect that this patient's obstructive jaundice and extrinsic biliary tree obstruction were caused by underlying cocaine use.

## Introduction

Cocaine is an illicit drug used by millions of people in the United States every day. The drug is extracted from the leaves of the Erythroxylum coca var plant and processed into benzoylmethylecgonine. Once the drug enters the body, it serves as a norepinephrine, dopamine, and serotonin reuptake inhibitor, causing psychosomatic effects [1,2]. It also behaves as a monoamine oxidase inhibitor and stimulates alpha receptors. Once chemically prepared, cocaine is usually adulterated with various substances, including pharmacologically inactive chemicals such as sugars and starches that can be purchased over the counter. It is also cut with pharmacologically active ingredients such as levamisole, caffeine, lidocaine, paracetamol/acetaminophen, diltiazem, and phenacetin to improve profitability. Notably, levamisole is the most common adulterant used. Some sources state that 69 -80% of the seized cocaine entering the United States contains levamisole [3]. The chemical levamisole pharmacologically functions very similarly to amphetamine and potentiates the effect of cocaine [1-3].

The primary side effects of cocaine are centered on the cardiovascular and nervous systems. It is well known that cocaine use can cause myocardial infarction, life-threatening arrhythmias, cerebrovascular accidents, and seizures. It has been documented to cause pulmonary complications such as pulmonary hypertension, pulmonary edema, pneumothorax, pneumomediastinum, pulmonary fibrosis, interstitial pneumonitis, and barotrauma. Other complications include retroperitoneal fibrosis, placental abruption, spontaneous abortion, and drug-induced psychosis [1-4]. The effects of cocaine on the gastrointestinal tract have been studied to a lesser extent, but studies have been limited to case reports and are rare. There are documented cases of cocaine-induced gastric and duodenal ulcer perforation, pancreatitis, intestinal ischemia, mesenteric ischemia, ischemic colitis, as well as small and large bowel perforation [1-7]. To the best of our knowledge, there are no documented cases of cocaine-induced biliary tree lymphadenopathy causing obstructive jaundice; however, we strongly believe that a chemical the cocaine was adulterated with caused periportal lymphadenopathy, resulting in extrinsic compression of the patient’s common bile duct. Thus, we feel such a clinical case scenario involving biliary obstruction in a patient that uses cocaine should include this differential diagnosis.

## Case presentation

**Figure 1:**
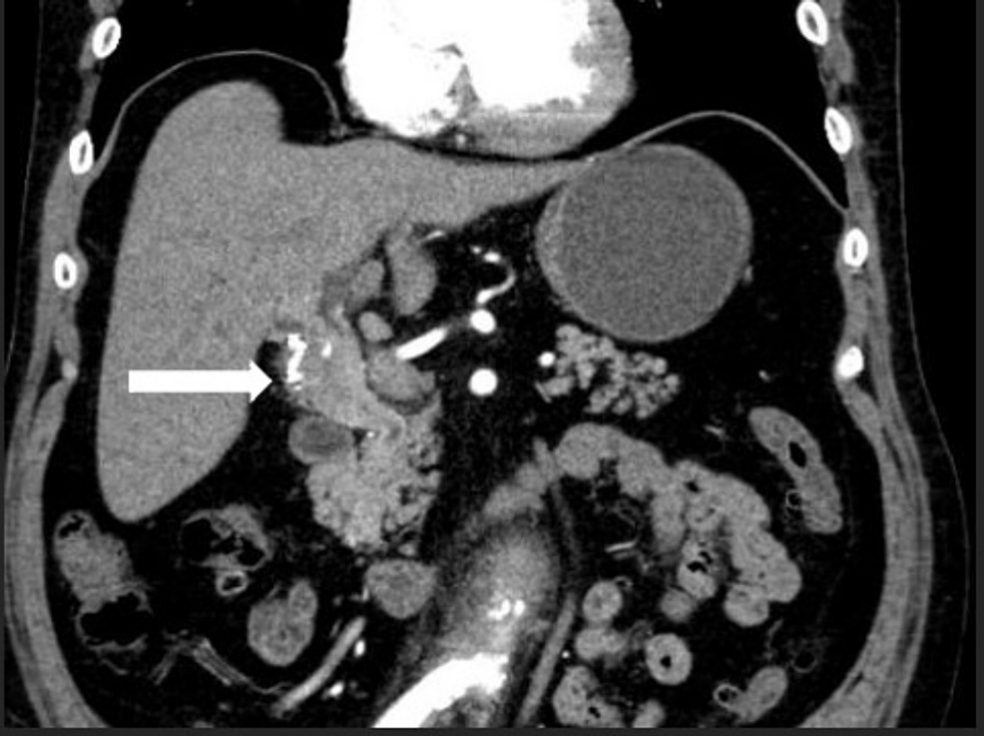
CTA of abdomen and pelvis with IV contrast coronal view White arrow pointing to a 3.1 x 2.8 cm mass compressing the common bile duct. CTA - computed tomography angiogram

**Figure 2:**
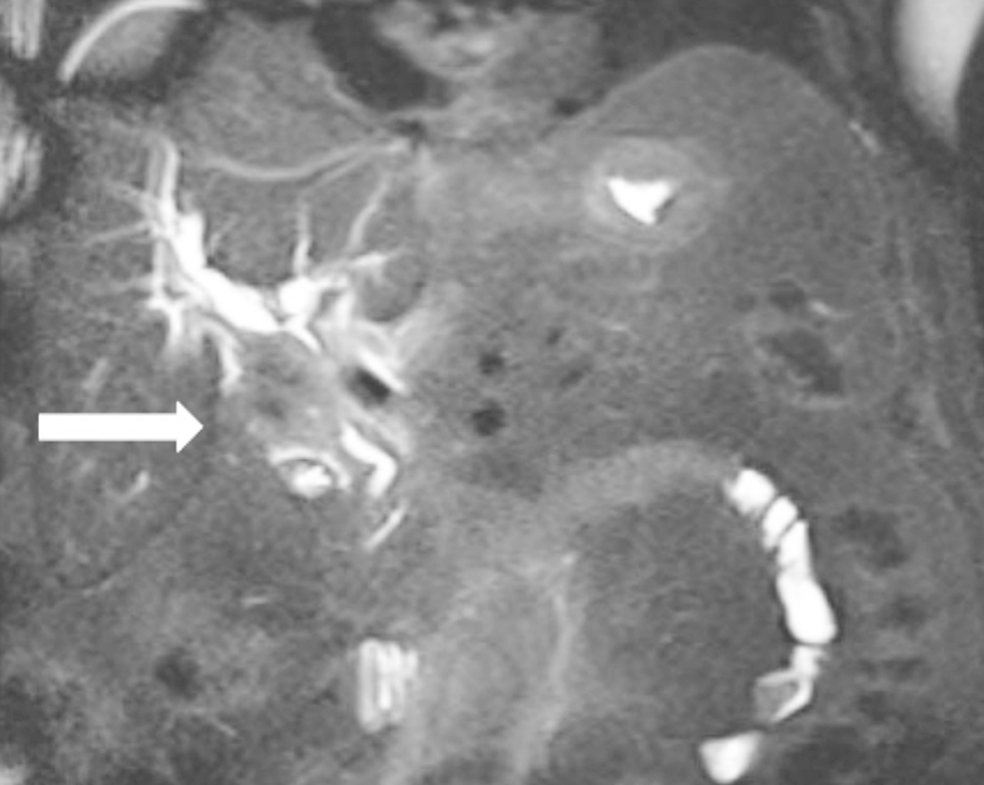
MRCP with IV contrast coronal view White arrow pointing to a 4.9 x 3.0 cm mass compressing the common bile duct. MRCP - magnetic resonance cholangiopancreatography

A 62-year-old male presented to the ED with abdominal pain, nausea, and vomiting. On physical examination, the patient had a soft abdomen with moderate epigastric tenderness and no scleral icterus or jaundice. The patient's electrolytes were within normal limits, the lipase was 27, the total bilirubin was 5.06, and the direct bilirubin was 3.6. He had a positive urinary drug screen (UDS) for cocaine on admission. An abdominal ultrasound was ordered, which initially showed a 1.5 cm dilated common bile duct with a surgically absent gallbladder. A computed tomography angiogram (CTA) of the abdomen and pelvis showed a 3.8 x 2.1 cm mass at the porta hepatis compressing the duct at that level (Figure 1). A magnetic resonance cholangiopancreatography (MRCP) showed a 4.9 x 3.0 cm mass at the porta hepatis with proximal biliary ductal dilation (Figure 2). A gastroenterologist was consulted, and an endoscopic retrograde cholangiopancreatography (ERCP) with stent placement and brush biopsy were performed, which showed stenosis of the proximal common bile duct suggestive of cholangiocarcinoma. Cytology results showed rare ductal atypical cells.

Our patient's bilirubin levels continued to rise despite the placement of a biliary stent, and interventional radiology was consulted for an internal-external drain biliary placement. Medical oncology was consulted as well, and given his imaging results, endoscopic ultrasound (EUS) was recommended on an outpatient basis. His total bilirubin and direct bilirubin normalized, and he was subsequently discharged with a prescription to obtain EUS and to follow up with our team and hematology and oncology. 

**Figure 3:**
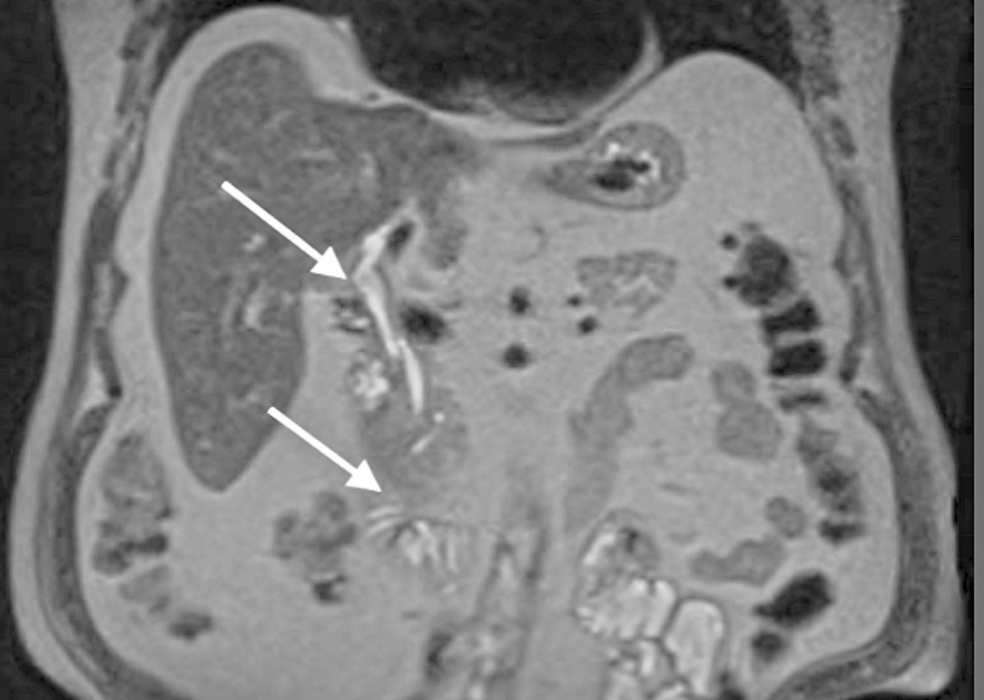
MRCP with IV contrast coronal view six months later White arrow showing no evidence of previously observed mass or signs of obstruction.

The patient had many social issues, including homelessness, drug addiction, and psychiatric issues. An EUS was performed, and the results were inconclusive, requiring the test to be repeated. He did not follow up to receive the repeat EUS and was admitted and discharged from our hospital several times over a span of six months for non-related issues. During his last admission, a repeat CT abdomen and pelvis showed no biliary obstruction or mass. The MRCP showed no ductal dilation, mass, or obstruction (Figure 3). Interventional radiology was consulted to perform a cholangiogram which showed no mass, obstruction, or dilation of the biliary tree, and the internal-external drain was removed. The patient was subsequently discharged in stable condition.

## Discussion

Cocaine can cause many different pathophysiological processes, including myocardial infarction, arrhythmias, pulmonary edema, pulmonary fibrosis, renal fibrosis, vasoconstriction, and intestinal ischemia [1-4]. Prior to being transported to the United States, cocaine is usually adulterated with various agents to increase profitability. These chemicals can include pharmacologically active and inactive chemicals such as sugars, starches, caffeine, phenacetin, lidocaine, benzocaine, acetaminophen, diltiazem, talcum powder, silica, calcium, quinine, cornstarch [3]. The most common compound used to adulterate cocaine is levamisole. The chemical was first produced as an antihelminth drug in the 1960s that works as the ganglion stimulant in mammals and a depolarizer muscular blocker in nematodes [3]. It was also used as an appetite suppressant but was withdrawn from the market for causing pulmonary vasoconstriction and hypertension. Levamisole is a chemical that functions similarly to amphetamines. It inhibits serotonin, dopamine, and norepinephrine reuptake and can potentiate the effects of cocaine [3]. The chemical has been found to cause agranulocytosis resulting in skin purpura, skin necrosis, ear necrosis, vascular thrombi, and vasculitis. Levamisole also has an inherent effect on patients that have coronary artery disease (CAD), as it can precipitate myocardial infarction, accelerate atherosclerosis, stimulate platelet aggregation and plaque rupture. In synergism with cocaine, it can exacerbate cocaine's vasoconstrictive properties [3].

Most cocaine entering the United States is already adulterated, then further adulterated with various chemicals upon being allocated to local distributors [7]. The chemicals previously mentioned in the article have been found to have significant pulmonary complications, including interstitial pneumonitis, fibrosis, pulmonary hypertension, alveolar hemorrhage, asthma exacerbation, barotrauma, thermal airway injury, hilar lymphadenopathy, and bullous emphysema [7]. There has not been a documented case of cocaine use causing biliary hilar lymphadenopathy, but there are studies that state adulterant chemicals such silica, talcum powder in cocaine have caused pulmonary hilar lymphadenopathy. We believe that one of the agents was admixed with the cocaine the patient abused and that it caused transient lymphadenopathy, which led to extrinsic compression of the common hepatic duct.

There have been other documented cases of biliary lymphadenopathy caused by abdominal tuberculosis. Abdominal tuberculosis is very rare, accounting for less than 1 % of the cases of tuberculosis. In the studies reported, the patients were young, ranging from 19-29 years of age [8-11]. They had symptoms of obstructive jaundice with elevated bilirubin levels. The diagnosis of hepatobiliary tuberculosis was made through tissue biopsy during exploratory laparotomy in one study. In another study, the authors were able to definitively make the diagnosis of hepatobiliary tuberculosis causing lymphadenopathy via a combination of laparoscopic ultrasound and fine-needle aspiration [8-11]. The patients were treated with a tuberculosis regimen of isoniazid, rifampin, ethambutol, and pyrazinamide. The patients did well after the disease process was correctly identified and appropriately treated. Our patient was never tested for tuberculosis, but extrinsic biliary obstruction resolved without any antimicrobial therapy [8-11]. He did not have any other symptoms of tuberculosis or abdominal tuberculosis, such as weight loss, anorexia, or low-grade fever. Although biliary lymphadenopathy induced by tuberculosis is possible, we do not believe this caused the patient's biliary extrinsic compression.

Sarcoidosis is a non-caseating granulomatous disease that has been shown to cause obstructive jaundice secondary to biliary lymphadenopathy in rare circumstances [12-13]. In those cases, the authors were able to obtain tissue diagnosis via ERCP biopsy. Studies have shown that 50- 79% of patients with sarcoidosis will also have liver involvement, but biliary tree involvement leading to obstruction is rare. Diagnosis and treatment involved ERCP with biopsy and stent placement. Patients responded very well to steroid taper. The liver function tests (LFTs) normalized, and they remained asymptomatic for up to 30 months post-treatment [13]. In our case, the patient did not have any history of sarcoidosis, but ERCP was performed with biopsy, which showed atypical cells with benign ductal epithelium. Our patient received stent placement but was never treated with steroids and his symptoms resolved. Biliary sarcoidosis remains on the list of differential diagnoses but, we do not think it is the etiology of our patient's biliary tree extrinsic compression.

Another theory behind our patient's unique pathology and the outcome is biliary stricture. Biliary stricture has many benign and malignant etiologies; however, it is more commonly associated with malignant etiology than with benign. Benign biliary stricture accounts for only 15-24% of cases [14-16]. Of the malignant causes, pancreatic adenocarcinoma is most common, followed by cholangiocarcinoma. Due to the fact that pancreatic adenocarcinoma is typically discovered in patients in the later stages, cancer has typically invaded the common bile duct by the time of detection, causing biliary obstruction [14-16]. Other malignant causes of biliary obstruction include primary duodenal adenocarcinoma, ampullary carcinoma, gallbladder carcinoma, hepatocellular carcinoma, lymphoproliferative disease, and metastatic disease [14-16]. Benign biliary stricture is most often caused by iatrogenic biliary injury during a cholecystectomy or as a result of an orthotopic liver transplant, which represents 80% of benign biliary stricture cases. Other causes include chronic pancreatitis, primary sclerosing cholangitis, IgG4-related disease, recurrent pyogenic cholangitis, HIV cholangiography, chemotherapy-induced sclerosing cholangitis, Mirizzi syndrome, and portal biliopathy [17-19]. 

Cocaine use has not been directly linked with extrinsic biliary tree obstruction, stricture, or constriction. The initial imaging results elevated total and direct bilirubin showing obstructive jaundice in our patient's case. His initial lipase was 27, making acute pancreatitis low suspicion for the cause of his symptoms. The patient's CT and MRCP did not show any pathology involving the pancreas, ruling out pancreatic cancers and chronic pancreatitis. Given the fact that our patient had a history of cholecystectomy, it is possible that he could have had a biliary stricture. Biliary stricture does not explain the fact that there was a mass visualized on MRCP and then the later resolution of the mass in question six months later on repeat MRCP [17-19]. The patient ultimately received an internal-external drain placed by interventional radiology, which would have treated the biliary stricture. We do not think the patient's biliary obstruction was caused by a biliary stricture.

There is a possibility that the patient's obstruction was caused by lymphadenopathy from lymphoma. In the case series written by Feller and Schiffman [9], they encountered two patients that had obstructive jaundice secondary to lymphoma. These patients were treated with nonoperative biliary bypass followed by chemotherapy and responded well to treatment [9]. In our case, the patient had no corrective therapy for lymphoma but had resolution of symptoms and signs of obstruction on multiple imaging modalities. Also, the patient did not have any other symptoms or physical exam findings consistent with lymphoma, such as lymphadenopathy, fevers, night sweats, weight loss, or suppressed appetite. Given this reasoning, we do not believe our patient's biliary obstruction was due to lymphoma or lymphadenopathy.

It is possible that the patient had a sphincter of Oddi dysfunction, which might have been exacerbated by cocaine use. In the study cited, a patient with hepatitis C and a history of the sphincter of Oddi dysfunction presented with dilated common bile duct after cocaine use [20]. The author believes that the common bile duct dilation was caused by cocaine exacerbating the patients' sphincter of Oddi dysfunction. In our case, the patient did not have a history of the sphincter of Oddi dysfunction. He also did not admit to any ongoing symptoms of this disease process during his multiple re-admissions. Cocaine-induced sphincter of Oddi exacerbation causing biliary obstruction remains low on the list of diagnoses explaining this rare case.

Overall, given the patient's use of cocaine and the exclusion of many other different types of pathology, we strongly believe the patient's biliary tree extrinsic compression, which was assumed to be cholangiocarcinoma at the time, was due to cocaine use. We spoke with a magnetic resonance radiologist at our institution who stated retrospectively the mass most likely represents biliary lymphadenopathy given its subsequent resolution on imaging six months later, but at the time, the more likely interpretation of the image was cholangiocarcinoma. From an extensive literature review to the best of our knowledge, this is the only report of cocaine-induced biliary lymphadenopathy.

## Conclusions

The case presented here is very rare and has many unique features. We strongly believe that our patient’s initial presenting pathology was related to an adulterant chemical used in the cutting process of cocaine causing biliary obstruction secondary to extrinsic compression from periportal lymphadenopathy. During the patient’s initial admission, our team was very close to treating the suspected obstruction surgically, but there were social issues complicated by psychiatric problems that precluded this. Repeat MRCP results six months later revealed resolution of the obstruction and mass. This case can be used as an excellent review and tool to widen the differential diagnosis of biliary obstruction secondary to extrinsic compression.
